# Minimally Invasive vs. Open Surgical Repair in Traumatic Diaphragmatic Hernia: A Systematic Review of 8,990 Patients

**DOI:** 10.7759/cureus.82371

**Published:** 2025-04-16

**Authors:** Erin E Major, Brian Chen, Ahmed D Al Mahrizi, Charles Ezenwanne, Harman Gill, Fatima Mossolem, Saameh A Siddique, Carlos Valladares

**Affiliations:** 1 Research, Futures Forward Research Institute, Toms River, USA; 2 Faculty of Medicine and Surgery, University of Malta, Msida, MLT; 3 Internal Medicine, RWJBarnabas Health, Toms River, USA

**Keywords:** diaphragmatic hernia, laparoscopic surgery, laparotomy, minimally invasive surgery, open hiatal repair, traumatic diaphragmatic hernia

## Abstract

Traumatic diaphragmatic hernia (TDH) occurs when abdominal contents herniate into the thoracic cavity, as the diaphragm is particularly susceptible to blunt or penetrating trauma. Currently, the standard treatments for TDH include minimally invasive surgical repair (MISR) and open surgical repair (OSR). MISR offers advantages such as reduced mortality, shorter hospital stays, and decreased postoperative pain and complications. In contrast, OSR is preferred for complex cases requiring better visualization, such as large defect repairs and comprehensive abdominal exploration. This study compares the clinical outcomes of MISR and OSR for TDH in adults, analyzing variables such as hospital stay, complications, recurrence rates, and postoperative pain. Following the 2020 Preferred Reporting Items for Systematic Reviews and Meta-Analyses or PRISMA guidelines, this systematic review was conducted across six online databases (PubMed, Embase, Scopus, Cochrane, Web of Science, and Google Scholar), screening 1,894 studies. Six comparative studies were included in the final analysis, comprising 8,990 patients (7,735 MISR; 1,255 OSR). Postoperative pain was measured using the visual analog scale (0-10), while hospital stays and recurrence rates were extrapolated from patient charts and reported as percentages. The results indicate that MISR offers potential benefits, including shorter hospital stays and fewer complications. Additionally, reduction in respiratory failure and postoperative pneumonia suggests improved hospital course with the minimally invasive approach. Comparable recurrence rates between techniques demonstrate that MISR achieves technical success similar to OSR when appropriately selected, with fewer complications and shorter hospital stays. In conclusion, MISR for TDH resulted in shorter hospital stays with fewer complications and had recurrence rates similar to OSR. However, variability in outcome reporting limits the clinical applicability of these conclusions and calls for further standardized studies.

## Introduction and background

**Figure 1:**
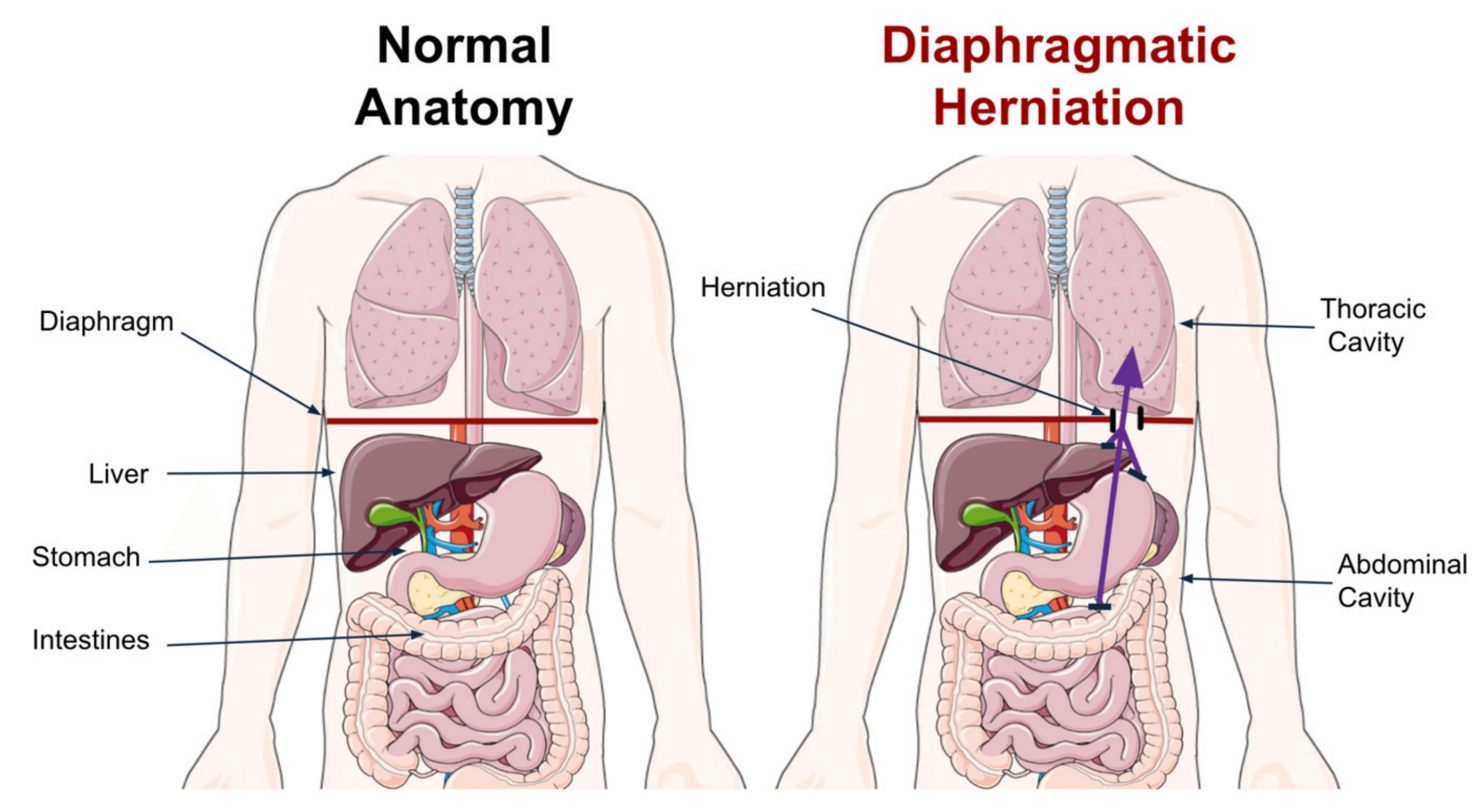
An illustration of normal thoracoabdominal anatomy (left) vs. traumatic diaphragmatic herniation (right), where abdominal contents migrate through a diaphragmatic defect into the thoracic cavity Image credit: The authors. This figure was partly generated using Servier Medical Art (http://smart.servier.com/), licensed under CC BY 4.0. Accessed: February 15, 2025.

Diaphragmatic herniation occurs when abdominal organs protrude through a traumatic or congenital defect of the diaphragm into the thoracic cavity (Figure 1) [1,2]. As with any other muscle, the diaphragm is susceptible to congenital defects, penetrating and blunt trauma, all of which can carry serious consequences [3-5]. Disruption of the diaphragm can lead to life-threatening complications such as respiratory difficulties or ischemic strangulation of the organs in the thoracic and abdominal cavities [3,4,6]. This is especially true in polytrauma patients where traumatic diaphragmatic hernia (TDH) is one of many possible internal injuries [3,4,6]. As TDH has been associated with elevated mortality, an emphasis has been placed on minimizing morbidity and preventing mortality through prompt diagnosis and effective surgical management [7-9]. Achieving a consensus on the most effective treatment approach for TDH has remained challenging, especially with the new dawn of robotic-assisted surgical technology [10]. However, there is a general agreement that surgical repair is currently the standard management for TDH, possessing two primary approaches: minimally invasive surgical repair (MISR) and open surgical repair (OSR) [4].

Most minimally invasive approaches have been increasingly used for TDH through laparoscopic and thoracoscopic methods [11]. Laparoscopic and thoracoscopic approaches use minimal-length incisions through which cameras and surgical tools are inserted, allowing surgeons to perform surgical repair without highly invasive incisions. As a result of these minimal incisions, MISR offers advantages such as decreased postoperative morbidity with shorter hospital stays, reduced postoperative pain, and fewer complication rates [12]. The MISR approach offers sufficient visualization and access to repair smaller or moderate-sized diaphragmatic defects [12-14]. However, its utility is somewhat limited in settings of complex patterns of injury, large herniations, and hemodynamic instability [12,15]. In these settings, an open surgical approach is preferred in acute trauma management [16]. OSR continues to be the method of choice for complex scenarios due to its ability to provide comprehensive inspection of the abdominal cavity, improved visualization of extensive injuries, and management of large defects [12-14]. However, in some cases, following complications, MISR is converted into OSR [12]. Although OSR is effective in repairing a wide range of TDH conditions, this approach has longer recovery times and an increased potential for postoperative morbidity [12-14]. Prior studies have indicated generally lower morbidity and shortened length of stay with MISR compared to OSR, but the small sample sizes and heterogeneity among included studies limit the ability to draw definitive conclusions [12]. High-quality evidence comparing the long-term outcome of MISR and OSR for TDH is still insufficient, which further strengthens the need for this review to present a summary of current knowledge and highlight specific areas in which future research is still necessary [12,13,17]. By synthesizing the available evidence, this study seeks to inform surgical decision-making and highlight areas for future research in TDH management.

This project was previously presented at the American College of Osteopathic Surgeons (ACOS) Winter Symposium on February 1, 2025.

## Review

Methods

This systematic review aims to compare the clinical outcomes of MISR and OSR for TDH in adults, focusing on hospital stays, complications, recurrence rates, and postoperative pain. Adhering to the 2020 Preferred Reporting Items for Systematic Reviews and Meta-Analyses or PRISMA guidelines, the review included a thorough search across six major databases: PubMed, Embase, Scopus, Cochrane, Web of Science, and Google Scholar. 

This systematic review used medical databases and gray literature to search for articles on the direct comparison of MISR to OSR in patients with TDH published between January 1, 2005 and January 1, 2025. On January 1, 2025, a search was conducted of PubMed, Embase, Scopus, Cochrane, and Web of Science using a focused search string, which can be further reviewed in the appendix.

Inclusion and Exclusion Criteria

**Figure 2:**
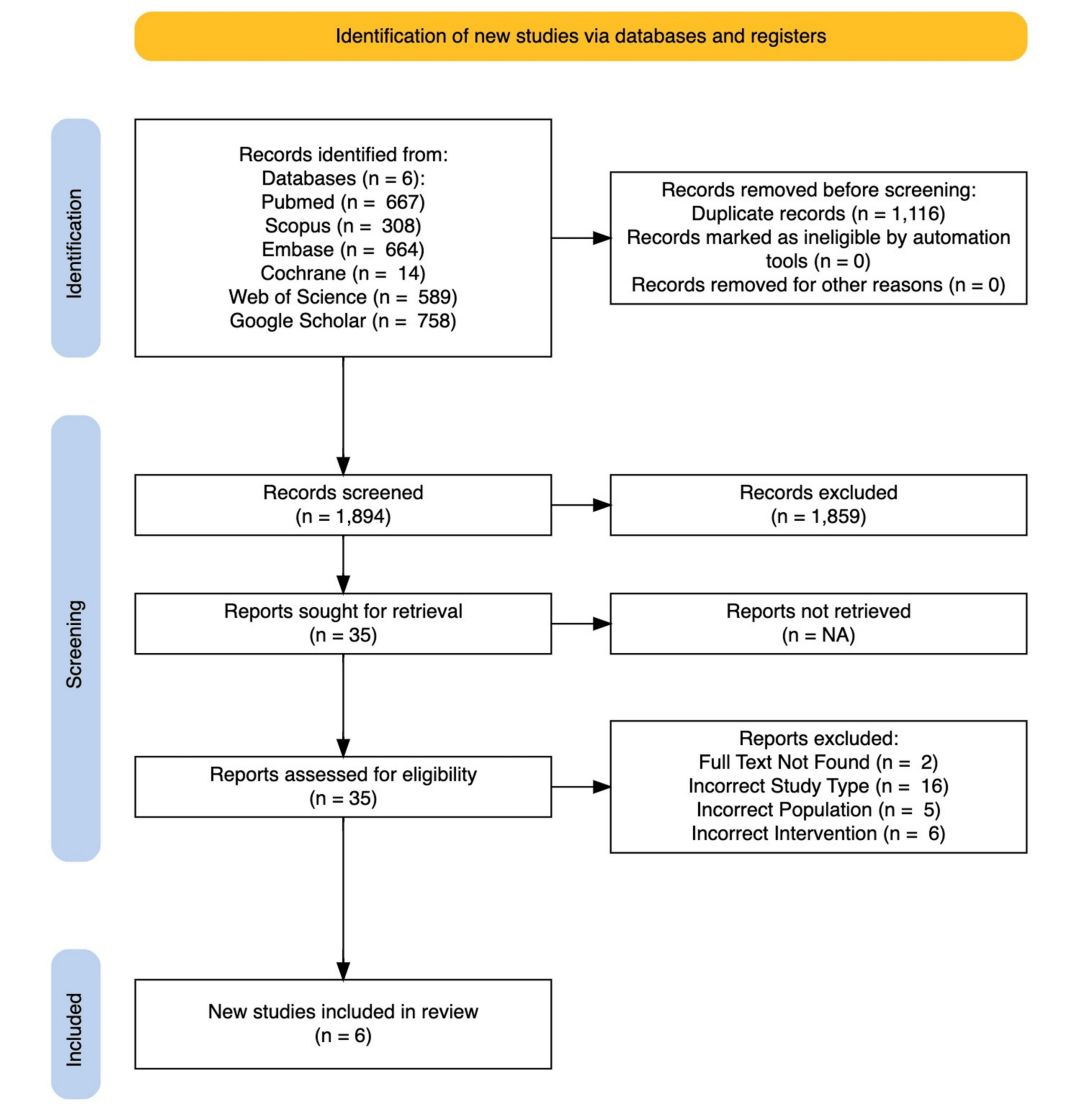
PRISMA flowchart illustrating the study selection process through the databases NA, not available; PRISMA, Preferred Reporting Items for Systematic Reviews and Meta-Analyses

The titles and abstracts of 3,010 studies were exported for screening [18]. Around 1,116 articles were removed as duplicates, leaving 1,894 studies to review (Figure 2). Titles and abstracts were independently screened and then included or excluded based on inter-rater agreement. This yielded 35 potential studies for full-text screening. Randomized controlled trials, prospective cohort studies, retrospective cohort studies, and case-control studies were eligible for inclusion. Included patients were ≥18 years old and diagnosed with TDH. Only MISR (laparoscopic or robotic) and OSR (laparotomy or thoracotomy) cases were included. Included papers also contained at least one of the following: operative time (in minutes), postoperative pain (measured by visual analog scale, 0-10), length of hospital stays (in hours), or recurrence rates (as a percentage, with specified follow-up period). Articles were also excluded if they were not in the English language and could not be translated.

ROBINS-IV2

In this review, the risk of bias was evaluated using the Risk Of Bias In Non-randomized Studies-of Interventions, Version 2 (ROBINS-IV2) framework, adapted for retrospective observational studies [19]. 

Results

Six studies involving 8,990 patients (7,735 MISR; 1,255 OSR) were eligible for data analysis and subjected to quantitative and qualitative data extraction (Figure 2). Heterogeneity and small sample size prevented meaningful analysis through meta-analysis, but quantitative analysis of mean difference in length of stay and risk ratio for recurrence rates is reported. 

Risk of Bias Assessment

**Figure 3:**
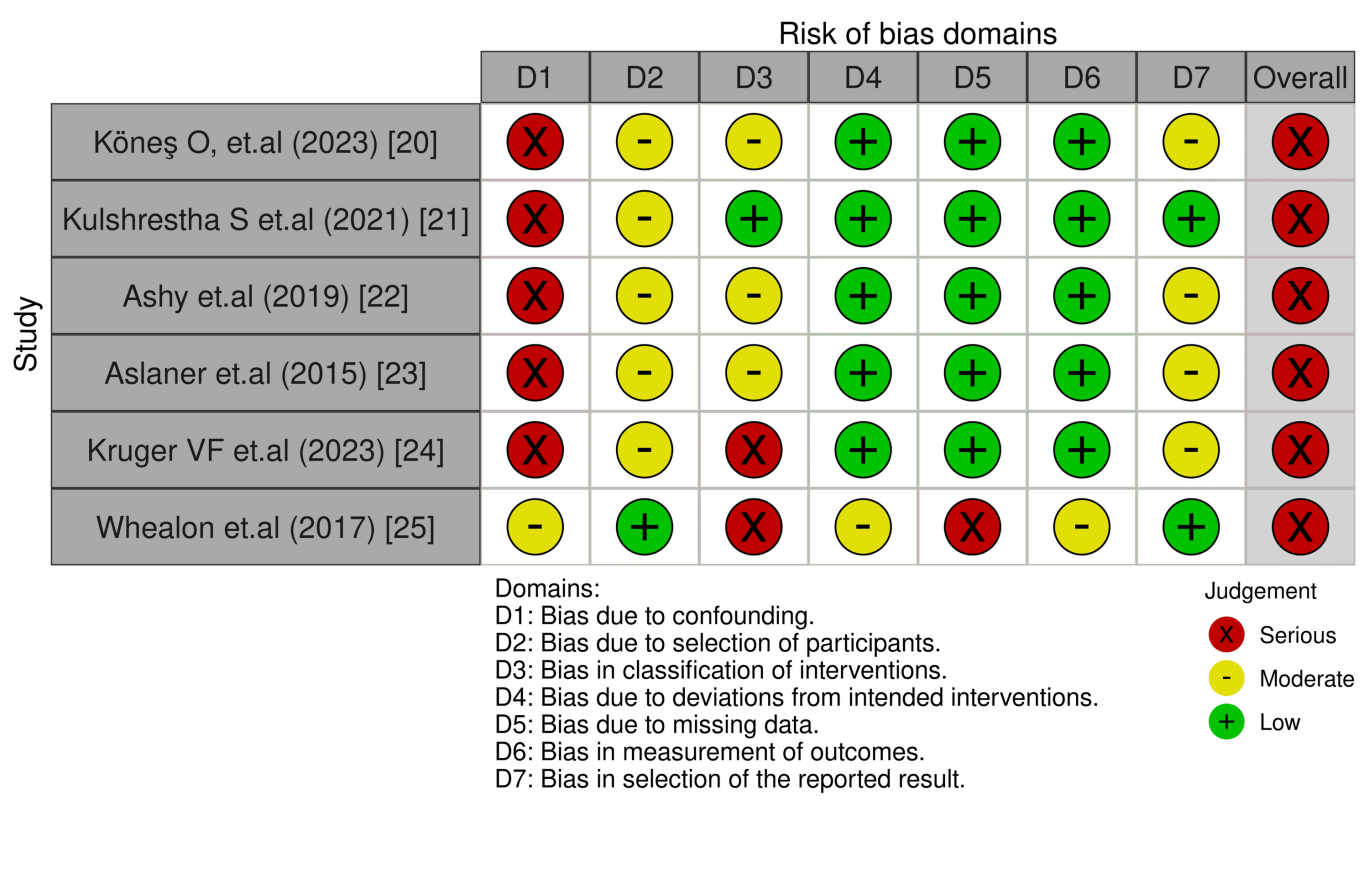
Traffic light plot of each risk of bias domain and overall risk of bias based on the ROBINS-IV2 guideline and algorithm [20-25] ROBINS-IV2, Risk Of Bias In Non-randomized Studies–of Interventions, Version 2

**Figure 4:**
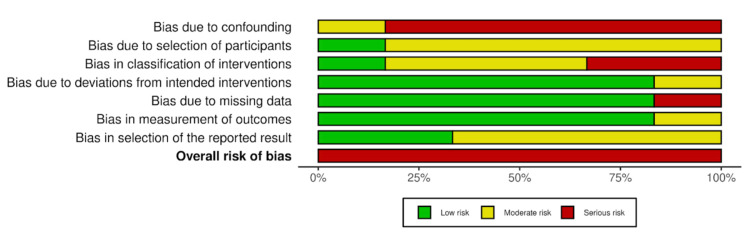
Summary plot of each risk of bias domain and overall risk of bias based on the ROBINS-IV2 guideline and algorithm ROBINS-IV2, Risk Of Bias In Non-randomized Studies–of Interventions, Version 2

The ROBINS-IV2 algorithm systematically assesses risk of bias in seven key domains (D1-D7). Results indicate heterogeneity in the methodological quality of the included study (Figures 3, 4). Whealon et al. exhibited a serious risk of bias due to intervention classification (D3) and missing data (D5). Kones et al., Kulshrestha et al., Ashy et al., and Aslaner et al. posed a serious risk of bias due to confounding variables (D1). Kruger et al. posed a serious risk of bias due to confounding variables (D1) and intervention classification (D3). According to the ROBINS-IV2 guidelines, one or more serious risk of bias in any of the seven key domains corresponds to a serious risk in the overall risk of bias (Figures 3, 4). None of the studies were identified to have a serious risk of bias due to selection of participants (D2), deviation from intended intervention (D4), measurement of outcomes (D6), and reporting of results (D7). According to the ROBINS-IV2, none of the studies were identified to have a critical risk of bias; therefore, they can still be included in the evidence synthesis. However, caution is warranted due to the overall serious risk of bias.

Hospital Stay 

Kones et al. was one of the included studies that did have tangible data comparing the hospital length of stay differences between open and minimally invasive TDH repair. The mean length of stay for MISR was 2 days as compared to 6 days in the OSR group [20]. Similar results were found in other included studies such as Kulshrestha et al. When comparing the hospital length of stay in this study, there was a mean length of stay of 2 days for the laparoscopic approach, and a mean length of stay of 3 and 6 days (p<0.001) for patients treated with robotic and open approaches, respectively (Table 1) [21]. Quantitative statistical analysis comparing OSR and MISR length of stay was conducted using data from Kulshethra et al. and Ashy et al. [22]. Analysis showed that hospital stay was significantly shorter in the MISR group by -0.40 days (95% confidence interval (CI): -0.62, -0.18, p<0.001). Aslaner et al. further support this difference. The study reported a cumulative mean length of stay for both open and laparoscopic approach of 10.2 days, which reflects a greater proportion of patients being treated with open compared to laparoscopic approach [23]. The overall finding of an increased length of stay for diaphragmatic hernia patients treated with OSR compared to MISR is further supported by the analysis of blunt trauma patients by Kruger et al. This study discusses how patients admitted for blunt injury, who primarily received open laparotomy, spent a considerably longer time on the trauma floor and intensive care unit [24]. Kruger et al. hypothesizes that this increased length of intensive care unit stay could be attributed to the higher rates of postoperative pneumonia in this group [24]. 

**Table 1:** Study characteristics and relevant results LOS, length of stay; MIS, minimally invasive surgery *Reported length of stay as cumulative for open and minimally invasive surgical approach

Study	Design	Total N	MIS N	Open N	MIS LOS (days)	Open LOS (days)	MIS recurrence	Open recurrence	Follow-up (months)
Köneş et al. (2023) [20]	Retrospective	70	42	11	*	*	2/70 (2.9%)	2/70 (2.9%)	81.6±35.16
Kulshrestha et al. (2021) [21]	Retrospective	8,858	7,338	1,520	2 (1, 4)	6 (4, 11)	-	-	12
Ashy et al. (2019) [22]	Retrospective	3	2	1	5	14	0/2	0/1	-
Aslaner et al. (2016) [23]	Retrospective	24	3	21	*	*	-	-	-
Kruger et al. (2023) [24]	Retrospective	12	1	11	-	-	-	-	-
Whealon et al. (2017) [25]	Retrospective	31,228	-	-	-	-	-	-	-

*Mortality Rates*

Another variable of interest essential to the assessment of outcomes of TDH repair patients is the rate of mortality. All included studies touched on mortality, with Kruger et al. reporting the highest rate. They reported that regardless of intraoperative or preoperative imaging diagnosis, patients who underwent MISR had a mortality rate of 16% [24]. Similarly, Whealon et al. found a higher mortality rate related to the volume of hospital admissions. Laparoscopic approach patients had an overall mortality rate of 0.14%, but there was a two-fold increase in mortality rates in lower volume admission hospitals [25]. This inverse relationship suggests that hospitals that admitted less patients had double the mortality rates compared to hospitals that admitted more patients. In contrast, Aslaner et al. found an overall mortality rate of 8% in the 24 eligible patients undergoing open and closed repairs, collectively [23]. Similarly, Kones et al. reported a total 30-day mortality rate of 7.1% in the 70 cases treated for TDH [20]. 

Postoperative Complications

Other complications of interest mentioned were the development of postoperative complications, such as pneumonia or wound dehiscence. A study by Kruger et al. reported the highest rates of postoperative pneumonia, with 50% of the blunt trauma patients developing pneumonia throughout their hospital course [24]. Quantitative statistical analysis of postoperative pneumonia and respiratory failure yielded a statistically significant difference between OSR and MISR. Respiratory failure was seen in 1.26% of OSR patients vs. 0.69% of MISR patients (p<0.001) [24]. Pneumonia was similarly seen in 1.27% of OSR patients vs. 0.64% of MISR patients (p<0.001) [24]. Kulshethra et al. also found that open approach patients had increased rates of wound complications [21]. Finally, similar to the rates of mortality, Whealon et al. found a statistically significant increase (p<0.001) in postoperative pneumonia in lower-volume hospitals [25]. 

Recurrence Rates

Another complication commonly seen in this patient population is the need for postoperative endoscopy. The rate of postoperative endoscopy was found to be statistically significant (p=0.04) between laparoscopic, open, and robotic approaches in the study by Kulshrestha et al. [21]. This study found that 2.1% of robotic repairs necessitated a return to the operating room for endoscopy compared to 1.8% in the laparoscopic group and 1% in the open repair group (p=0.04). The rate of return to the operating room for repeat repair, however, was statistically similar (p=0.84) among both groups in the study by Kulshrestha et al. Across all studies with reported data on recurrence rates, statistical analysis revealed no significant differences in either technique, with a risk ratio of 0.89 (95% CI: 0.37, 2.14, p=0.79).

Index Hospitalization Cost

Kulshrestha et al. followed patients admitted for robotic, laparoscopic, and OSR for a period of 12 months, allowing the study to evaluate the cost of index hospitalization [21]. Retrospective analysis showed that robotic and open surgical approaches were associated with a $5,268 and $6,282 (p<0.001) increase, respectively, in the cost of index hospitalization as compared to the laparoscopic group. 

Discharge Disposition 

Discharge disposition is another variable that is closely correlated with the overall outcome and postoperative quality of life in patients treated surgically for TDH repair. Kulshrestha et al. evaluated this variable and found that OSR patients were less likely to be discharged home and were instead more likely to be discharged to skilled nursing facilities as compared to those who underwent laparoscopic or robotic repair [21]. 

Discussion

The overall findings of this systematic review suggest that MISR, specifically the laparoscopic approach, is cost-effective, has a shorter length of hospital stay and decreased mortality during index hospitalization [26]. Analysis further suggested that there were no differences between techniques with respect to recurrence rates. Postoperative pain and operative time were inconsistently reported throughout the reviewed papers. As such, operative time and postoperative pain results provided less clinically significant interpretation. There were, however, decreases in mortality, hospitalization cost, complication rates and lower acuity discharge disposition associated with MISR. 

**Figure 5:**
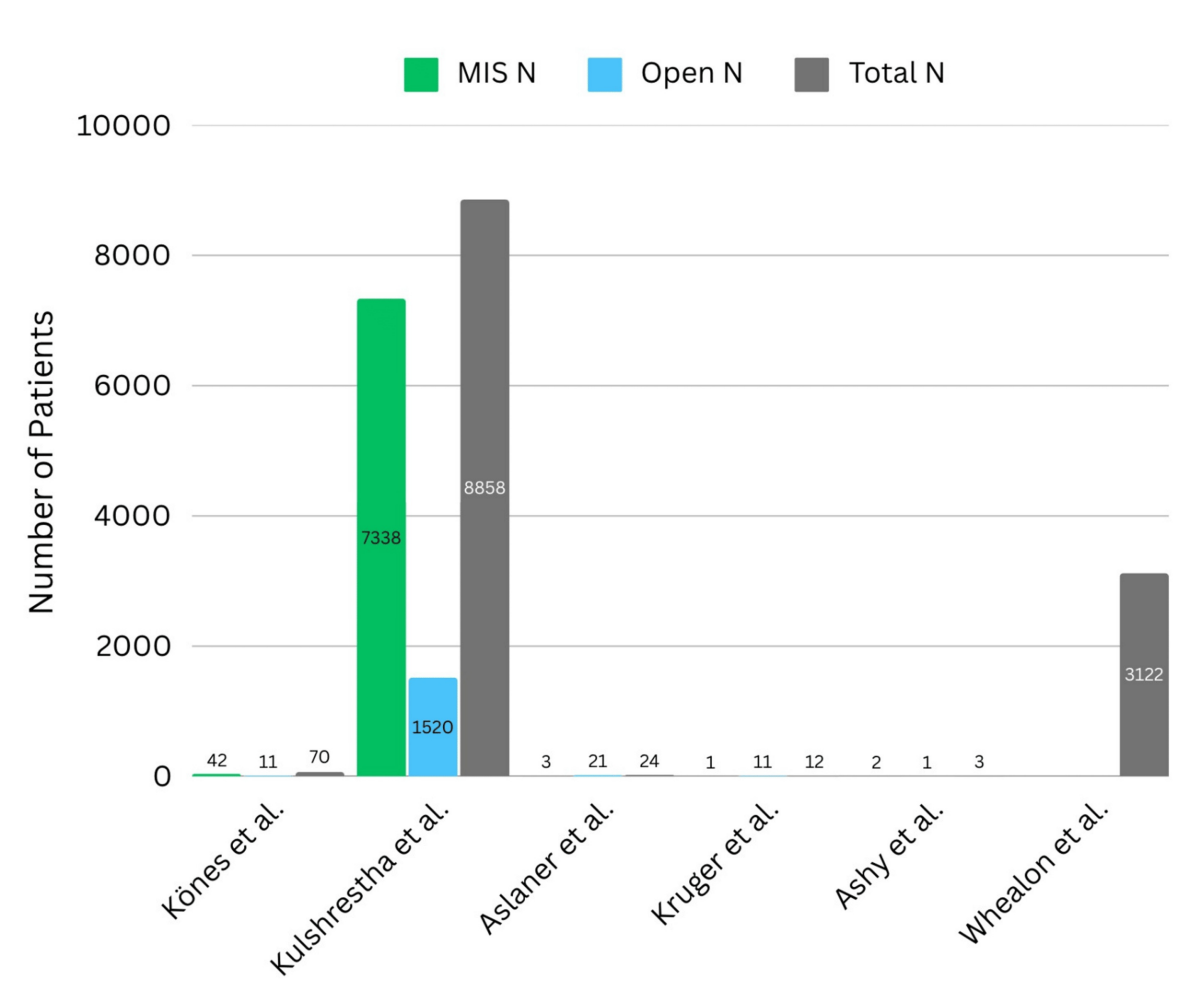
Number of patients for minimal invasive surgery (MIS N), open surgery (open N), and unreported (total N) [20-25]

While studies collectively reported lower mortality rates associated with MISR, these values warrant critical evaluation. For example, Aslaner et al. reported a total mortality of 8%, but the study had a significantly greater proportion of patients who received OSR (87.5%) compared to MISR (12.5%) (Figure 5). With a skewed representation of surgical technique in these patients, the total mortality rates in this study are less reflective of a pure laparoscopic, or minimally invasive, approach. In contrast, Kones et al. reported a total 30-day mortality of 7.1%, with 60% of patients undergoing a laparoscopic technique. With the greater percentage of patients receiving the laparoscopic approach, this study should be considered more reflective of the true mortality rate associated with minimally invasive laparoscopic repair. Furthermore, the finding of decreased mortality rate associated with MISR warrants greater discussion. The high mortality rate associated with OSR may be delineated by the association of critically ill patients with higher grade herniation requiring an open approach to address the inherently greater severity of the injury [27]. Unwanted but not uncommon consequences of OSR include longer healing times and longer hospital stays that can predispose patients to hospital-acquired infection, which could account for the differences in these variables between OSR and MISR [28,29]. With this consideration in mind, further studies should aim to assess if the higher mortality rate commonly associated with OSR is correlated with injury severity scores. 

Hospitalization cost is another data point that varied across included studies, with MISR associated with the lowest cost of index hospitalization [26]. Minimally invasive approaches, such as laparoscopic, could be useful in the setting of limited resource hospitals where robotic surgical devices may not be available or when high costs associated with robotic devices limit their use [30,31]. MISR also suggested superiority, as the rates of postoperative pneumonia and wound complications were found to be lower with this approach as compared to OSR [21,24,25]. Finally, OSR patients were more likely to be discharged to a skilled nursing facility than to be discharged home, suggesting that discharge disposition for MISR typically required lower-acuity post-discharge care [21].

While the results of this study are invaluable in the application to clinical practice, they are not without limitations. First, it is important to note that not all included studies isolated direct comparisons of MISR and OSR. The lack of direct comparison, in addition to the inconsistent reporting of sought out variables of interest, provides weaker evidence to support the findings of this review. Furthermore, vast differences in sample size between studies may limit the generalizability of the results. Moreover, despite an extensive search of over 2,000 studies, only six comparative studies met our inclusion criteria, highlighting the limited available data. This scarcity of high-quality, well-designed studies underscores a significant gap in the literature regarding optimal surgical treatment approaches for TDH [17].

Current research suggests MISR for TDH may offer benefits such as reduced hospital stays and fewer complications. However, these findings remain inconclusive, particularly due to the variability in reporting and small sample sizes. The lack of long-term outcome data further complicates the ability to draw definitive conclusions [12,13]. Given the clinical significance of TDH and its associated complications, there is an urgent need for more robust, standardized studies comparing MISR and OSR. 

## Conclusions

Overall, the findings of this study report that the length of stay for minimally invasive approaches, such as robotic and laparoscopic procedures, was significantly shorter than traditional open surgical approaches. As of February 2025, this is the most recent systematic review to exclusively evaluate the outcomes of MISR in comparison to OSR, especially in adult patients. MISR is suggested to offer significant benefits, including minimal postoperative complications and reduced hospital stays; however, further research is required to substantiate these findings. Once again, the lack of available data on long-term outcomes, recurrence rates, and quality of life after surgery are significant examples of the remaining understudied effects in this population, highlighting an extensive gap in the literature. Given the lack of available literature on this subject, it is suggested that future research addresses the limitations identified in this review to provide clearer guidelines for TDH management and improve clinical effectiveness.

## Appendices

Search String: 

(diaphragm* OR diaphragmatic) AND (hernia* OR rupture* OR injur*) AND (trauma* OR accident*) AND (surg* OR repair* OR operat*) AND (laparoscop* OR thoracoscop* OR minimally invasive OR open). On the same date, a secondary search was conducted on Google Scholar using the search string: ("traumatic diaphragmatic hernia" OR "traumatic diaphragmatic rupture") AND (laparoscopic OR thoracoscopic OR "minimally invasive") AND (repair OR surgery) AND (comparison OR "versus open"). 
